# Metavirome Sequencing of the Termite Gut Reveals the Presence of an Unexplored Bacteriophage Community

**DOI:** 10.3389/fmicb.2017.02548

**Published:** 2018-01-04

**Authors:** Chinmay V. Tikhe, Claudia Husseneder

**Affiliations:** Department of Entomology, Louisiana State University Agricultural Center, Baton Rouge, LA, United States

**Keywords:** metavirome, termite, bacteriophages, symbiosis, endosymbionts

## Abstract

The Formosan subterranean termite; *Coptotermes formosanus* is nutritionally dependent on the complex and diverse community of bacteria and protozoa in their gut. Although, there have been many studies to decipher the taxonomic and functional diversity of bacterial communities in the guts of termites, their bacteriophages remain unstudied. We sequenced the metavirome of the guts of Formosan subterranean termite workers to study the diversity of bacteriophages and other associated viruses. Results showed that the termites harbor a virome in their gut comprised of varied and previously unknown bacteriophages. Between 87–90% of the predicted dsDNA virus genes by Metavir showed similarity to the tailed bacteriophages *(Caudovirales)*. Many predicted genes from the virome matched to bacterial prophage regions. These data are suggestive of a virome dominated by temperate bacteriophages. We predicted the genomes of seven novel Caudovirales bacteriophages from the termite gut. Three of these predicted bacteriophage genomes were found in high proportions in all the three termite colonies tested. Two bacteriophages are predicted to infect endosymbiotic bacteria of the gut protozoa. The presence of these putative bacteriophages infecting endosymbionts of the gut protozoa, suggests a quadripartite relationship between the termites their symbiotic protozoa, endosymbiotic bacteria of the protozoa and their bacteriophages. Other than Caudovirales, ss-DNA virus related genes were also present in the termite gut. We predicted the genomes of 12 novel *Microviridae* phages from the termite gut and seven of those possibly represent a new proposed subfamily. Circovirus like genomes were also assembled from the termite gut at lower relative abundance. We predicted 10 novel circovirus genomes in this study. Whether these circoviruses infect the termites remains elusive at the moment. The functional and taxonomical annotations suggest that the termites may harbor a core virome comprised of the bacteriophages infecting endosymbionts of the gut protozoa.

## Introduction

Bacteriophages are considered as the most abundant biological entities on earth with their total number estimated to be 10^31^ (Wommack and Colwell, 2000; Suttle, 2005). Bacteriophages play an important role in the ecosystem by carrying out nutrient recycling via bacterial cell lysis (Wilhelm and Suttle, 1999; Rodriguez-Brito et al., 2010; Jasna et al., 2017). In the marine ecosystems bacteriophages have been shown to be responsible for metabolic reprograming (Hurwitz et al., 2013; Hurwitz and U'Ren, 2016). Bacteriophages also carry out horizontal gene transfer (Eggers et al., 2016; Touchon et al., 2017) and are considered as a driving force behind bacterial genome evolution (Canchaya et al., 2004; Zeng et al., 2017). Along with the horizontal gene transfer, bacterial-phage antagonistic co-evolution is hypothesized to have a major impact on bacterial and bacteriophage diversification (Buckling and Rainey, 2002; Paterson et al., 2010). Previous studies have shown that the majority of the differences observed in the genomes of different bacterial strains of the same species in the human gut were related to restriction-modification systems, and glycosyltransferases, both of which play a key role in phage defense (Zhu et al., 2015). Bacteriophage-resistant strains have been shown to possess modifications in their surface receptors, including-antigens and outer membrane proteins (Bassford et al., 1977; Labrie et al., 2010). These surface receptors also have an important role in cell surface adhesion (Shin et al., 2005) and thus may alter the interactions of bacterial strains with their host (Lerouge and Vanderleyden, 2002). Co-evolution of bacteriophages and bacteria has been shown to alter the metabolic capacities of bacteriophage-resistant strains including the ability to utilize certain carbon sources (Middelboe et al., 2009). All these observations show that phage-bacterial interactions can have a profound effect on the ecology of the microbial community and can in turn affect the host harboring the community.

Termites rely on their symbiotic gut microbial community for cellulose digestion and acquisition of essential nutrients (reviewed in Brune, 2014). The microbial community of the higher termites (family: Termitidae) is comprised of bacterial symbionts while the lower termites (Rhinotermitidae, Mastotermitidae, Hodotermitidae, Kalotermitidae, Termopsidae, Serritermitidae) harbor flagellated protozoa in addition to the bacterial symbionts. Workers of the Formosan subterranean termite (FST), *Coptotermes formosanus* (Family: Rhinotermitidae), harbor three species of protozoa in the hindgut paunch that are essential for the survival of the termite colony (Eutik et al., 1978). The worker caste specializes in providing nutrition for the entire termite colony by digesting lignocellulose, and the gut protozoa enhance the endogenous cellulolytic capability of the worker gut. The protozoa also harbor endo- and ecto-symbiotic bacteria which carry out nitrogen fixation and amino acid production (Hongoh et al., 2008; Desai and Brune, 2012). The termite gut microbial community is responsible for many complex biochemical processes, providing the termite host with essential nutrients. In a previous study based on cloning of 16S rRNA gene amplicons, at least 213 different bacterial ribotypes were reported from the gut of the FST (Husseneder et al., 2010). A closely related species, *Coptotermes gestroi*, was estimated to harbor a bacterial community of 1,460 different species using next-generation sequencing (Do et al., 2014). The termite gut presents a unique scenario in which the host is heavily dependent on the microbial community and any changes to this community can potentially have detrimental effects on the host (Rosengaus et al., 2011). The presence of a multifaceted complex bacterial community makes the termite gut a perfect ecological niche for the presence of a diverse bacteriophage community. Like many other ecosystems the extent of this diversity and the impact of bacteriophages on the microbial community in the termite gut remains unstudied.

Bacteriophages have been previously shown to be associated with the gut bacteria in higher termites (Ottesen et al., 2006). We previously isolated and sequenced the first bacteriophage from a termite hindgut. This phage, CVT22, infects *Citrobacter* sp. found in the gut of the FST (Tikhe et al., 2015). Two additional novel bacteriophages, Tyrion and Arya (NC_031077.1 and NC_031048.1) infecting *Enterobacter* sp. were also isolated and sequenced from the termite gut (manuscripts under review). In a recent study by Pramano et al. a bacteriophage infecting “Candidatus *Azobacteroides pseudotrichonymphae*” was discovered, making it the first bacteriophage to be associated with an obligate intracellular mutualistic endosymbiont (Pramono et al., 2017). These reports of novel bacteriophages in the termite gut indicate the presence of an unexplored bacteriophage community. The main obstacle in studying the bacteriophages from the termite gut using conventional isolation techniques is the non-culturable bacterial community (Hongoh, 2010). Most of the bacteria from the termite gut are not culturable in the lab using conventional methods, which makes the study of their bacteriophages difficult. Bacteriophages also lack universal marker genes. It has been shown that virome sequencing can be used as a powerful tool to study and characterize viral communities (Sullivan, 2015). In recent years, advances in the field of marine viromics has established a foundation for all the other virome studies (Brum and Sullivan, 2015). Many marine virome studies have identified enormous diversity of previously unknown protein sequences whose functional roles still largely remains unknown (Roux et al., 2015b; Brum et al., 2016). Metavirome sequencing also circumvents the requirement of isolation and, therefore, we chose this approach to study the bacteriophage community in the FST gut in its entirety.

In this study we report the virome sequencing of the guts of workers from three FST colonies with the intention of exploring the unstudied bacteriophage diversity. This study is the first effort focused entirely on uncovering the bacteriophages and any other possible viruses associated with a termite species. Studying the virome of the termite gut will help us to understand the complex quadripartite relationship between the termite host, protozoa, bacteria symbionts, and associated bacteriophages.

## Materials and methods

### Termite collection

Workers of the FST were collected from three different colonies in New Orleans, Louisiana, USA, using untreated in-ground bait stations. Termite colonies were designated as Colony 1 (collected from City Park, on 06/21/2013), Colony 2 (collected from Hayne Blvd., lakefront on 06/18/2013), and Colony 3 (collected from Cypress St. on 06/21/2013). The distance between each of the three colonies was more than 9 km, which by far exceeds the swarming distance of the winged reproductives (alates) of the FST (Messenger and Mullins, 2005) thereby ensuring that the three colonies were founded independently by unrelated alates. All the termites were brought back to the lab in a plastic container containing a moist filter paper. Termites were processed immediately for viral community DNA extraction.

### Extraction of viral community DNA

A total of 500 worker termites from each colony were dissected and their guts were suspended in 3 ml sterile phosphate buffered saline (pH 7.5) kept on ice. The guts were homogenized vigorously using a sterile pestle until a uniform solution was formed. The homogenate was centrifuged at 10,000 g and the supernatant was filtered twice through a 0.22 μm syringe filter. The filtered homogenate was then treated with 2.5 units per μl of RNase A and DNase I at 37°C for 6 h. The filtrate was then mixed with 200 μl of 0.5 M EDTA, and DNA was isolated using phenol-chloroform-isoamyl alcohol extraction. Concentration and quality of the extracted DNA was checked with NanoDrop® ND1000. Bacterial DNA contamination was checked via PCR using 27f and 1492r universal 16S rRNA gene primers (Lane, 1991). The initial concentrations of the extracted DNA from Colony 1, Colony 2, and Colony 3 were 4, 1.8, and 2.5 ng/μl, respectively. The extracted DNA was amplified using illustra GenomiPhi V2 DNA Amplification Kit (GE Healthcare Life sciences, Pittsburgh, USA). The amplified DNA was then ethanol precipitated and dissolved in sterile distilled water.

### Next generation sequencing and bioinformatics analysis

From each of the three colonies, 50 ng of DNA was used to prepare the libraries using Nextera DNA Sample Preparation Kit (Illumina). The insert size of the libraries was determined by Experion Automated Electrophoresis Station (Bio-Rad). The insert size of the libraries ranged from 300 to 850 bp (average 500 bp). Individual libraries were sequenced at Molecular Research LP, Shallowater, Texas, on the Illumina MiSeq platform (Colony 1: 2 × 250 bp, Colony 2 and Colony 3: 2 × 300 bp). Quality of the DNA reads was checked using FASTQC (Andrews, 2010). DNA reads were checked for Illumina adaptor contamination and reads below the Phred score of 20 were removed using Trim Galore (Krueger, 2015). DNA reads were assembled into contigs using SPAdes Genome Assembler (Version 3.0) available on the Illumina BaseSpace platform with the default parameters using error correction and assembly mode (Bankevich et al., 2012). The contigs obtained were uploaded on the Metavir server for taxonomic assignments of the predicted ORFs using RefSeq complete viral genome protein sequence database from NCBI (released on 01/11/2017) (Roux et al., 2014). All the predicted protein coding genes were also blasted against the protein sequences from bacteriophage ProJPt-1Bp1 (Pramono et al., 2017). The contigs were also analyzed using VirSorter to separate viral contigs from cellular contaminants (Roux et al., 2015a). Contigs available on Metavir were screened for the presence of *VP1, TerL*, and *Rep* genes. For the construction of phylogenetic trees, we used full length amino acid sequences of terminase large subunit TerL (terminase_1, terminase_6, terminase_3, terminase_GPA, and terminase_1), Microviridae VP1, and Circoviridae Rep proteins. The sequences were aligned using MUSCLE (Edgar, 2004). Maximum likelihood trees were constructed using PhyML algorithm with a WAG substitution model (Guindon et al., 2010). For *Microviridae* subfamily assignment, full-length amino acid sequences of VP1 protein from Quaiser et al. (2015) were used to construct a phylogenetic tree. Contigs containing *VP1, TerL*, or *Rep* genes were analyzed manually for the presence of other putative viral genes. Contigs were classified as of a viral origin using the parameters described previously, with the POG13 database was used instead of POG10 (Bellas et al., 2015). Putative partial or full phage genomes were annotated manually and comparative genomic diagrams were generated using Easyfig (Sullivan et al., 2011). Putative viral genomes were visualized using CGview (Grant and Stothard, 2008) and SnapGene® (from GSL Biotech; available at snapgene.com). PHACTS analysis was carried out to determine the lifestyle and host of the putative phages (McNair et al., 2012). Family assignment of putative phage genomes was performed with VIRFAM using the ACLAME database (Lopes et al., 2014). For contig LSPY100002, RNA polymerase beta and beta' subunit sequences from phiKZ-like bacteriophages were used to construct phylogenetic trees (Lavysh et al., 2016). Functional annotation was carried out using the MG-RAST automated pipeline with an integrated M5NR database (Keegan et al., 2016). Orthologous genes from the three colonies were compared against each other using Orthovenn (Wang et al., 2015). All the assembled contigs have been submitted to NCBI GenBank under the accession numbers LSPY0000, LSQA0000, and LSPZ0000. Fully annotated contigs from this study are publicly available on the Metavir server under the study named “termite gut metavirome” (http://metavir-meb.univ-bpclermont.fr).

## Results and discussion

### DNA extraction, next generation sequencing, and bioinformatics analysis for taxonomic assignment

Worker termites from three different termite colonies were used for this study. The abundance and diversity of microbes in the worker termites' gut was the biggest challenge in separating the viral community DNA from all other microbial DNA to avoid contamination. High speed centrifugation and double filtration through 0.22 μm were carried out to remove any contaminating bacterial and protozoal cells along with other bigger particles. Other free contaminating nucleic acids in the filtrate were degraded by RNase and DNase and DNA was extracted from the filtrate. No bacterial contamination was detected by PCR with universal 16S rRNA primers. At this stage the amount of DNA to be used for next generation sequencing was extremely low. In many virome studies, multiple displacement amplification (MDA) has been used to increase the amount of DNA required for sequencing (Minot et al., 2011; Santiago-Rodriguez et al., 2015; Tangherlini et al., 2016). MDA, however is known to introduce bias in the DNA samples with preferential amplification of ssDNA viruses (Kim and Bae, 2011). While the presence of viruses presented in this study is undisputed, their relative abundance may not represent the actual viral composition in the termite gut. Many new methods are being established to overcome the bias of amplification techniques and present absolute quantitate estimation of viral communities (Roux et al., 2016).

This DNA was then submitted for next generation sequencing using Illumina MiSeq platform. The sequencing data, predicted genes by Metavir and MG-RAST, and the number of circular contigs predicted by Metavir are summarized in Table 1.

**Table 1:** Sequencing data, gene prediction, and number of circular contigs from viral DNA isolated from the guts of the FST workers from three different colonies.

	**Colony 1**	**Colony 2**	**Colony 3**
# raw reads	2,693,057^a^	1,670,422^b^	1,293,080^b^
# contigs (pre NCBI/MG-RAST QC)	4,413	10,539	9,440
N50/N75	5,157/1,216	4330/1000	3202/949
# contigs (post NCBI/MG-RAST QC)	4,347	10,022	9,190
Largest contig	251,606	299,025	246,064
GC (%)	41 ± 9	45 ± 10	40 ± 9
# genes predicted (Metavir)	9,497	22,389	21,850
# predicted proteins (MG-RAST)	6,523	14,282	14,723
# of circular contigs	79	104	132

a *Illumina MiSeq platform (2 × 250 bp)*,

b *Illumina MiSeq platform (2 × 300 bp)*.

Metavir assigned 27.13% of the genes from Colony 1, 27.85% from Colony 2 and 28.9% from Colony 3 as virus affiliated genes with an e-value of 10^−5^ or less. Compared to Metavir, MG-RAST classified fewer genes as viral. Percentage of genes classified as viral for Colony 1 was 9.57%, for Colony 2 was 3.8%, and for Colony 3 was 3.53%. Taxonomically MG-RAST classified between 79 and 93% of the genes as bacterial. The alpha diversity calculated by MG-RAST for Colony 1, 2, and 3 was 551, 598, and 231 species respectively. The difference between the taxonomic assignments by MG-RAST and Metavir has been previously observed in the metavirome sequencing of Antarctic soils. These viromes were predicted to be dominated by temperate bacteriophages (Zablocki et al., 2014). The taxonomic assignment of phage DNA as bacterial is likely due to the fact that reference databases classify prophages as bacterial when they are integrated into a bacterial chromosome at the time of genome sequencing. To further analyze if our data was contaminated with bacterial cellular contaminants bearing prophages, we used VirSorter tool, which can effectively separate bacterial and viral contigs (Roux et al., 2015a). VirSorter did not predict prophage sequences of category 1, but it did predict a total of 14 contigs (0.0005%) as prophage-like (category 2 and category 3). These results suggests a minimal contamination by bacterial genomes containing prophages. The number of sequenced bacterial genomes is reaching the 100,000 mark while the bacteriophage genomes still remain poorly represented in the NCBI database (around 2000 *Caudovirales* genomes, as of 06/01/2017). Sequencing more phage genomes is paramount to improve recognition of prophage sequences in bacterial genomes and will improve taxonomic assignments in all virome studies.

Previous studies showed that Bacteroidetes form around 70% of the bacterial flora of the guts of *C. formosanus* workers (Noda et al., 2005; Shinzato et al., 2005). For this reason, we expected the Bacteroidetes phages to dominate the virome composition. The virome composition however, was different from what we expected, with Proteobacteria and Firmicutes comprising at least 40% of the identified genes (MG-RAST). This difference could be explained by the small number of sequenced Bacteroidetes bacteriophages in reference databases. Since we sequenced filtrate from the termite gut it was not surprising that viral sequences were at least 42 times enriched in our data as compared to the unfiltered metagenome of a higher termite, *Nasutitermes* sp. (Warnecke et al., 2007). Functional annotation also showed that phage-related sequences were at least 10 times enriched in our data. The overall enrichment in phage-related genes and PCR results indicate a successful separation of bacterial contaminants during viral DNA purification.

**Figure 1:**
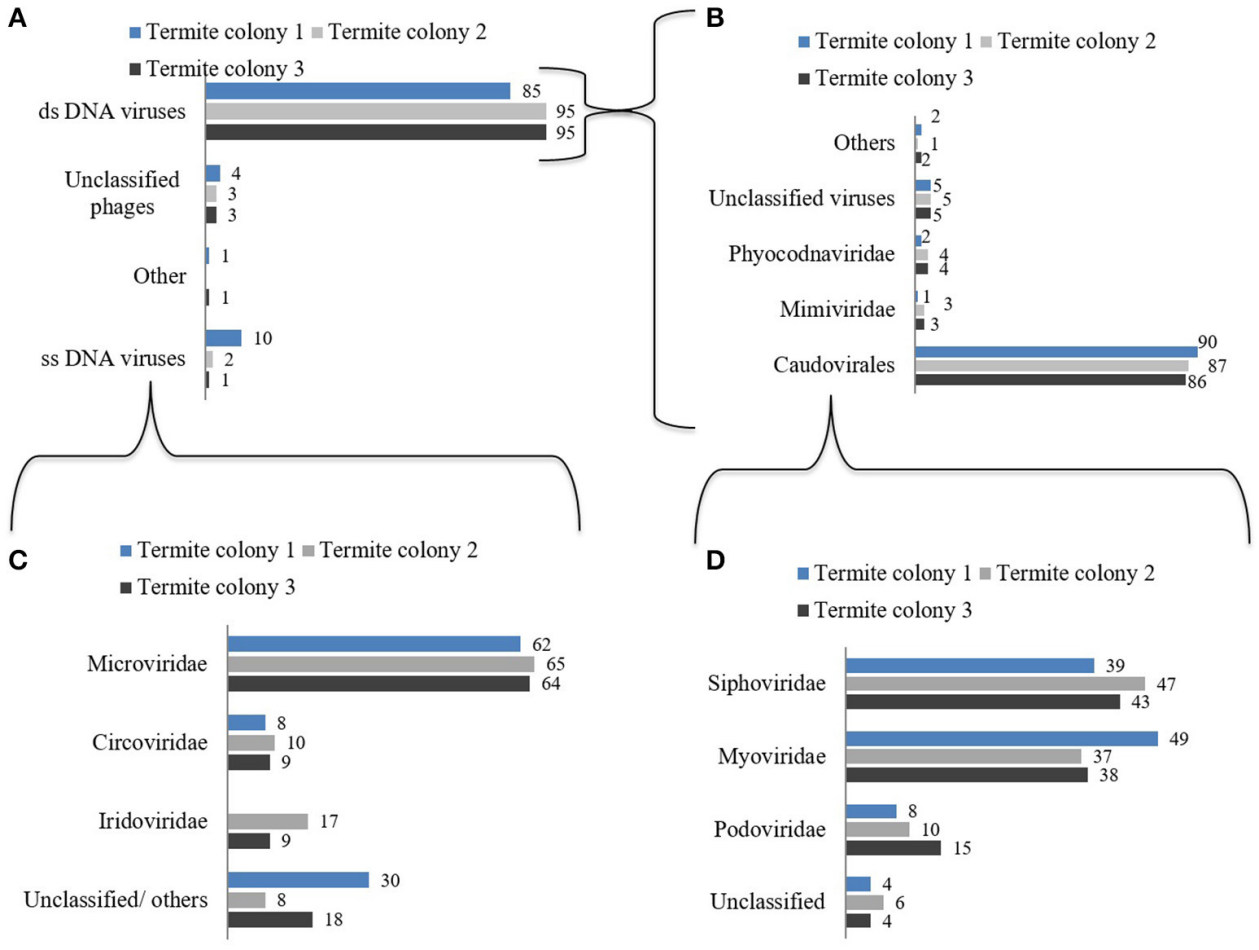
Classification and abundance (%) of various virus types observed in the guts of the termite workers from three different colonies. **(A)** Percent of taxonomic assignment of all viral genes **(B)** Percent of taxonomic assignment of dsDNA virus related genes. **(C)** Percent taxonomic assignment of ssDNA virus related genes. **(D)** Percent taxonomic assignment of *Caudovirales* related genes. The data were generated using Metavir-2 server by comparing the predicted proteins to the NCBI virus protein database. Only top BLAST hits with an e-value of 10^−5^ or less were used (normalized for each category).

Taxonomic assignment using Metavir showed that dsDNA virus-related genes were dominant amongst all the viral genes despite the MDA amplification and its likely bias towards ssDNA viruses (Figure 1A). The dsDNA viral genes predominantly belonged to the tailed bacteriophages from the order Caudovirales (Figure 1B). Genes related to all three families of the order *Caudovirales*, i.e., Myoviridae, Siphoviridae, and Podoviridae were present in all three termite colonies (Figure 1D). Apart from the genes related to Caudovirales, genes related to large eukaryotic dsDNA viruses and other unclassified viruses were also present in all the three termite colonies. The single stranded DNA viruses (ssDNA) contributed between 1 and 10% of the total virus related genes. Within the ssDNA virus related genes between 62 and 65% belonged to Microviridae phages (Figure 1C).

### Gene based diversity of tailed bacteriophages

**Figure 2:**
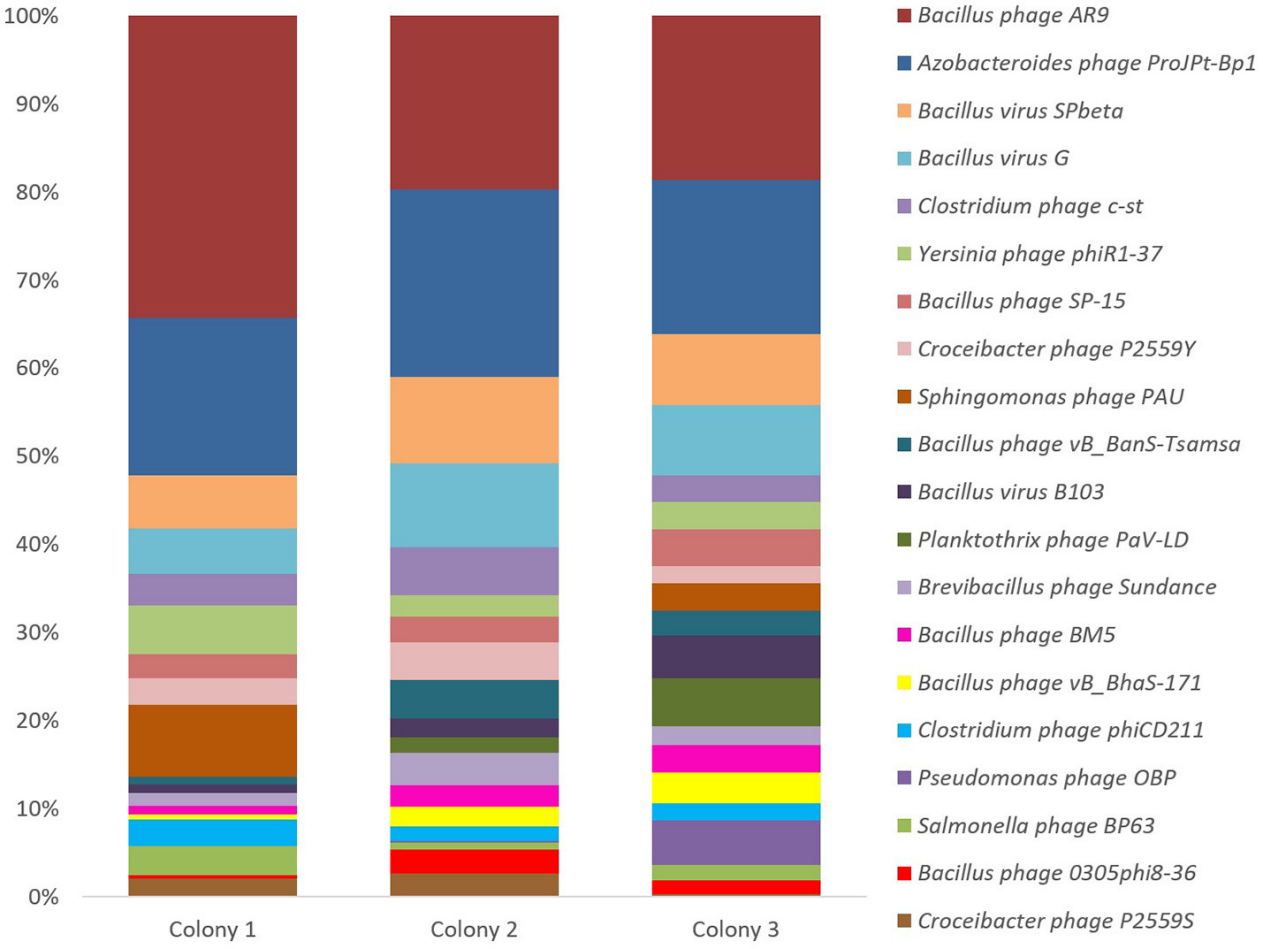
Taxonomic distribution (%) of the predominant dsDNA bacteriophage genes from the guts of termite workers from three different colonies (normalized). The data were generated using Metavir-2 server by comparing the predicted proteins to the NCBI virus protein database. Only top BLAST hits with an e-value of 10^−5^ or less were used.

Among all three termite colonies, Colony 3 was the most diverse in terms of tailed bacteriophages, with genes related to 712 different bacteriophages, followed by Colony 2 (598 bacteriophages) and Colony 1 (389 bacteriophages). Genes related to a total of 960 different tailed bacteriophages were observed across all three termite colonies. This number accounts for 48.65% of all tailed bacteriophages whose complete genomes are available in the NCBI Genbank database (04/12/2017). Of these 960, Siphoviridae-related bacteriophages were the most diverse, representing 483 different bacteriophages, followed by Myoviridae (335) and Podoviridae (142). A considerable number of bacteriophages (218) were shared by all three termite colonies, with *Bacillus* phage AR9 (Lavysh et al., 2016) and *Azobacteroides* phage ProJPt-1Bp1 (Pramono et al., 2017) related genes being present in the highest proportions (Figure 2). In Colony 1, 10% of all the classified sequences belonged to *Bacillus* phage AR9 followed by Colony 2 (6%) and Colony 3 (4%). *Azobacteroides* phage ProJPt-1Bp1 related genes constituted 6.45% of all the classified genes in Colony 1, 5.61% in Colony 2 and 3.92% in Colony 3. Of the top 20 most dominant tailed bacteriophage related genes from all the three termite colonies, 12 bacteriophages had Firmicutes as their host out of which 9 bacteriophages were infecting *Bacillus* spp.

The analysis of phage diversity of the three termite colonies suggests the presence of a conserved virome of tailed bacteriophages in the termite gut from the same geographical area. Although there is some degree of inter-colonial variation, nearly a quarter of all tailed bacteriophages (23%) were present in all three colonies. This hypothesis needs to be tested by a larger study including more colonies from the introduced and native distribution range of the FST (Husseneder et al., 2010).

### Phylogenetic analysis of terminase genes

**Figure 3:**
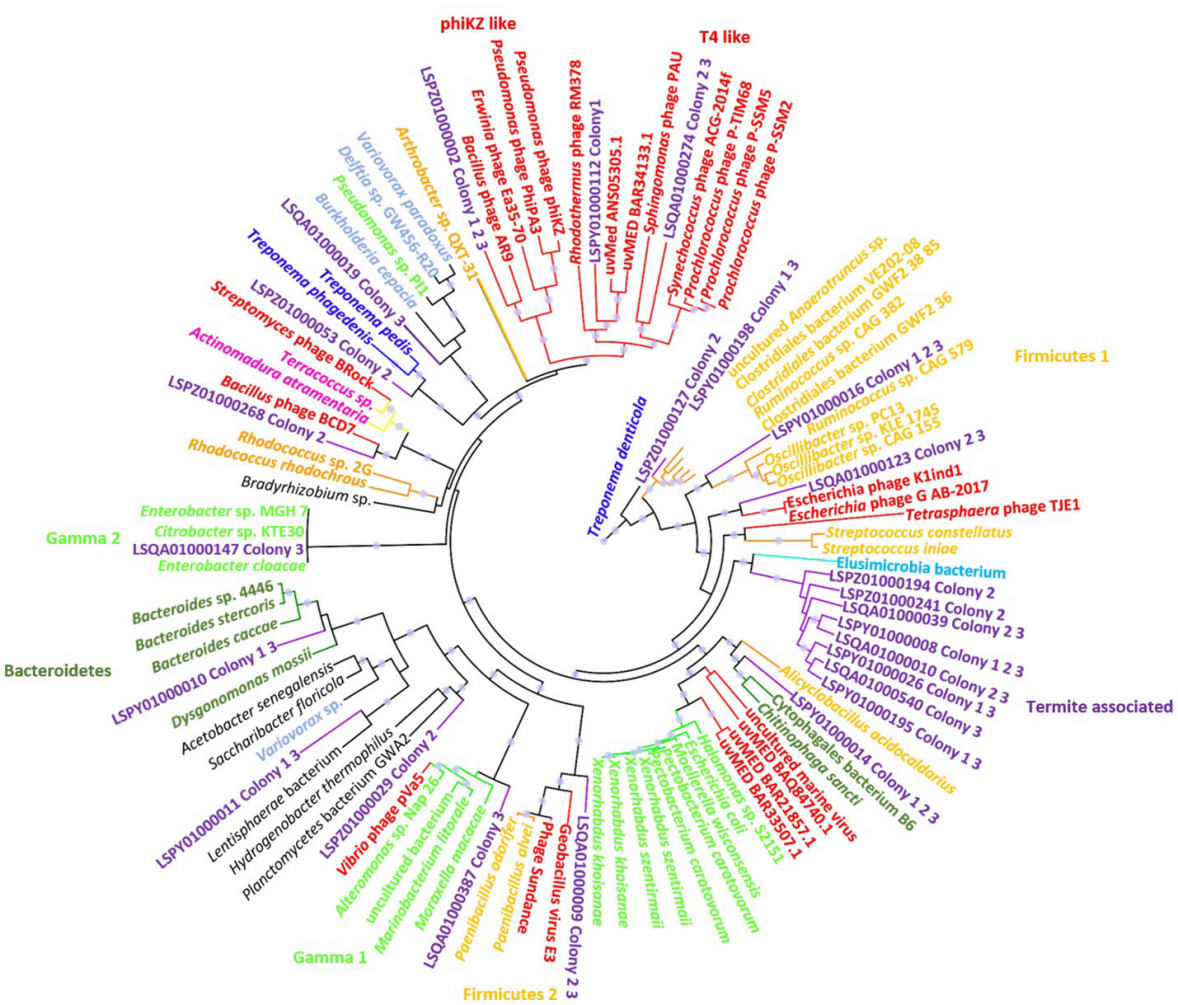
A maximum likelihood phylogenetic tree of large terminase subunit of type terminase_6. The nodes with a bootstrap value of 70% or more are indicated by a circular symbol. Sequences from the termite gut are colored purple. Bacteriophages: red. Firmicutes: orange. Spirochetes: dark blue. Gammaproteobacteria: bright green. Bacteroidetes: dark green. Actinomycetes: pink. Alphaproteobacteria: light blue. Termite Group I bacterium: sky blue. Others: black.

From all three termite colonies together, 51 unique full length terminase large subunit amino acid sequences were predicted. Out of the 51 sequences, 25 contained terminase_6 (pfam03237) domains, 12 contained terminase_3 (pfam04466.8) domains, 9 had terminase_gpa (pfam05876) domains, and 5 had terminase_1 (pfam03354) domains. Our data of terminase diversity in the termite gut are comparable to the results from virome sequencing of the deep sea, where 52 unique terminase sequences were identified (Mizuno et al., 2016). Most of the terminase sequences from the termite gut matched closely to the terminase genes from prophage regions in the bacterial genomes (Figure 3).

Phylogenetic analysis of terminase_6 showed a vast diversity with matches to prophages associated with a wide range of bacterial taxa. These results were also similar to the deep sea sequencing data, where terminase_6 domain was the most abundant type (Mizuno et al., 2016). Eight terminases from the virome formed a separate clade comprising a terminase gene from a Termite Group 1 bacterium. Two terminase genes were clustered with two separate Firmicute clades, another two were clustered with two separate Gammaproteobacteria clades, and one was clustered with a Bacteroidetes clade. Another clade was comprised entirely of bacteriophages from the Myoviridae family and three genes from termite gut phages. The clade was further subdivided in phiKZ-like bacteriophages and T4-like bacteriophages (Figure 3). Remaining genes were present in multiple clades comprised of terminases from the genomes of taxonomically diverse bacterial phyla (putative prophages).

Phylogenetically, terminase_3 genes were divided into four clearly separated clades. The Alphaproteobacteria clade and the Bacteroidetes clade each contained three terminases associated with the termite gut virome, while both the Enterobacteriaceae and the Firmicute clade contained one. Two other terminases found in the termite gut virome formed separate branches while one was grouped with *Clostridium* sp. CAG 306 (Figure S1). Several subunits of terminase_GPA from the termite gut virome were assigned to two distinct clades of Spirochetes and Alphaproteobacteria. The Spirochetes cluster contained five terminase_GPA subunits from the termite gut while another subunit formed a sister clade with Alphaproteobacteria (Figure S1). Most of the terminase_1 subunits were associated with Firmicutes or bacteriophages infecting the Firmicutes and one sequence was clustered with Bacteroidetes (Figure S1).

The phylogenetic analysis of the terminase genes indicated that most of the genes matched to prophage regions in the bacterial genomes rather than sequenced bacteriophage genomes. These results along with MG-RAST annotations and VirSorter analysis suggest that most of the termite gut bacteriophages might be temperate in nature.

### Phylogenetic analysis of the integrase genes

The integrase gene is used by the temperate bacteriophages to enter the lysogenic life cycle. It has been shown that prophage integrates in the host genome with a minimum impact on the overall chromosomal architecture. The bacteriophage also undergoes numerous adaptations according to the host genome in order to establish a lysogenic life cycle (Brüssow et al., 2004). It can be assumed that temperate bacteriophages are likely to infect closely related bacteria or bacteria where the overall genome architecture is conserved. Thus, phylogenetic analysis of phage integrase is likely to yield more information about its host. A total of 31 unique phage integrase sequences were identified from the three termite colonies. Phylogenetic analysis of phage integrase genes showed that sequences from the termite gut are clustered with a wide range of bacterial taxa (Figure S2). Five termite gut integrases were clustered within a Spirochete clade, six were associated with Firmicutes, another six were distributed in two clades comprised of Spirochetes and Bacteroidetes, and four were associated in two clades comprised of Bacteroidetes and Firmicutes. The remaining genes were distributed in clades comprised of diverse bacterial phyla. The results suggest the presence of temperate bacteriophages capable of infecting all the major bacteria taxa in the termite gut.

### Putative contigs of dsDNA bacteriophage origin

We compared individual contigs to Refseq virus, Pfam-A, POGs13, and ACLAME database as described previously (Bellas et al., 2015). Many contigs were considered to represent putative complete or partial bacteriophage genomes. The details of all the contigs described below are listed in Supplementary Table 1.

### LSPY01000004 and LSPY01000006 represent genomes of bacteriophages infecting the symbiotic bacteria of the gut protozoa

**Figure 4:**
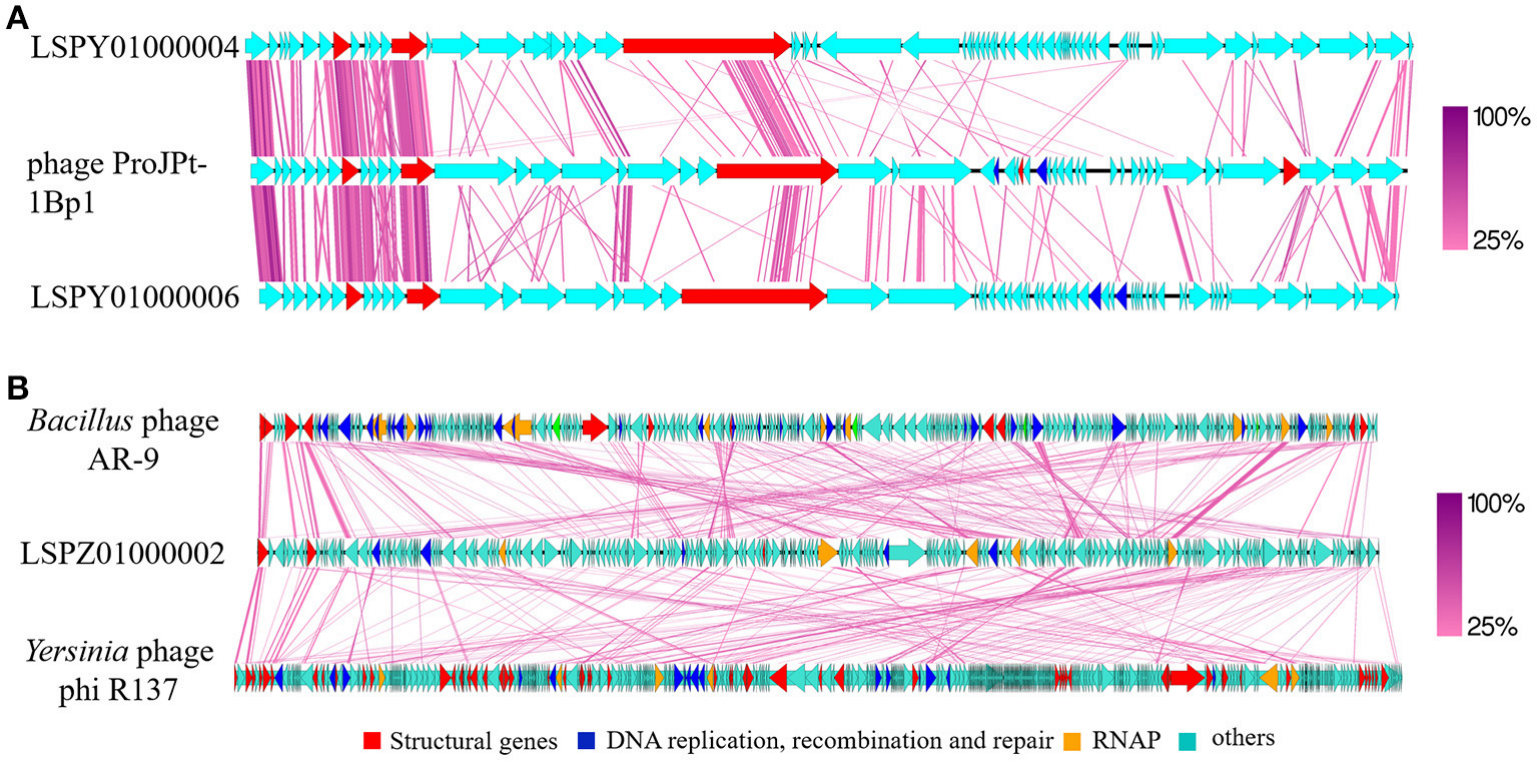
**(A)** Comparative genomic analysis of LSPY01000004 and LSPY01000006 with *Azobacteroides* phage ProJPt-1Bp1. All three circular genomes have been rearranged so that the start codon of a conserved hypothetical protein is the first base in the sequence. **(B)** Comparative genomic analysis of LSPZ01000002 with *Bacillus* phage AR9 and *Yersinia* phage phi R137. All three circular genomes have been rearranged so that the start codon of the large terminase subunit is the first base in the sequence. The figures were generated using Easyfig software with tblastx. The structural genes are indicated in red, DNA metabolism related genes are indicated in blue and RNA polymerase genes are indicated in orange. All the other genes are indicated in sky blue color.

Both LSPY01000004 and LSPY01000006 were predicted as circular contigs in termite Colony 1. Generally, circular contigs are indicative of a complete genome. Contigs mapping onto LSPY01000004 and LSPY01000006 were present in all the three termite colonies, suggesting an inter-colonial conserved distribution. Out of the 68 predicted genes in LSPY01000004, 30 produced a match in NCBI nr protein database with an e value of 10^−5^ or less. Of those 30, 21 genes matched only to *Azobacteroides* phage ProJPt-1Bp1, a bacteriophage infecting an obligatory intracellular bacterium Candidatus *A. pseudotrichonymphae* of the termite gut protozoa (Pramono et al., 2017). Out of the remaining genes, five genes matched to two different plasmids from Ca. *A. pseudotrichonymphae* and one gene matched to the genome (Hongoh et al., 2008). A total of 65 genes were predicted in contig LSPY01000006, of which 36 produced a match in the NCBI database. Out of those 36, 22 matched to phage ProJPt-1Bp1, 7 genes matched to plasmid pCFPG3 from Ca. *A. pseudotrichonymphae* and 1 gene matched to the genome of Ca. *A. pseudotrichonymphae*. There was very little similarity at nucleotide level in the genomes of LSPY01000004 and LSPY01000006 (73% match over 5% of the genome); most of the similarity was observed in the region of conserved hypothetical proteins also found in the genome of ProJPt-1Bp1. Overall, the genome arrangement of LSPY01000004, phage ProJPt-1Bp1, and LSPY01000006 was alike with areas of high similarity and synteny (Figure 4A). LSPY01000004 and LSPY01000006 showed many differences in the hypothetical proteins. Notably, LSPY01000006 harbored a gene similar to dihydrofolate reductase (DHFR) which was absent in LSPY01000004 and phage ProJPt-1Bp1. T4 bacteriophage DHFR has been predicted to play an important role in DNA metabolism and was also predicted to be a part of the virion particle (Mosher et al., 1977). However, at this moment the function of DHFR in bacteriophages remains to be studied.

VIRFAM analysis of ProJPt-1Bp1, LSPY01000004, and LSPY01000006 predicted them to be the members of *Caudovirales*; however, the contigs could not be assigned to any family. The genome of phage ProJPt-1Bp1 was captured while sequencing the host bacterial genome and a t-RNA detected in the phage genome also matched to the host (Pramono et al., 2017). We were not able to identify any t-RNA in LSPY01000004 and LSPY01000006 or any other genes, which could point toward the potential host of these phage-like genomes. However, based on the similarity of genes to phage ProJPt-1Bp1 and Ca. *A. pseudotrichonymphae*, LSPY01000004 and LSPY01000006 most likely infect Ca. *A. pseudotrichonymphae* or a closely related symbiont of the obligatory gut protozoa. Also, it should be noted that phage ProJPt-1Bp1 was sequenced from Ca. *A. pseudotrichonymphae* from the gut of *Prorhinotermes japonicus* from Japan collected in 2012. It has been demonstrated for Ca. *A. pseudotrichonymphae* that their protozoa hosts and the termites have co-diversified (Noda et al., 2007; Ikeda-Ohtsubo and Brune, 2009; Desai et al., 2010). This presents us with a possibility of co-diversification of bacteriophages along with their hosts. The observed differences and the conserved genome architecture between phage ProJPt-1Bp1, LSPY01000004, and LSPY01000006 can be explained by the bacteriophage co-diversification hypothesis. A study of more similar phage genomes from various termite species will shed more light on the association and co-diversification of bacteriophages, protozoa, their symbiotic bacteria, and the termite host. One interesting feature that was notable in LSPY01000004, LSPY01000006, and ProJPt-1Bp1 is the apparent absence of conserved phage genes like terminase, endolysins, and phage integrases. According to the best of our knowledge, no Caudovirales bacteriophage without the presence of a terminase gene has been found prior to our study. There are two explanations for the missing genes in these phages. One is that these bacteriophages have lost their signature genes and are maintained in the bacteria as plasmids. Whether this relationship is symbiotic, as hypothesized in the case of phage ProJPt-1Bp1 (Pramono et al., 2017), remains to be explored. The other possibility is that these types of bacteriophages have a completely new method of carrying out essential functions such as DNA packaging and host lysis. Gene expression analysis would answer the question whether these bacteriophages are dormant or play any role in the bacterial metabolism, or enter an infective cycle.

In this study, we centrifuged the gut homogenate and also filtered it through a 0.22 μm to remove all possible bacterial contamination. The free nucleic acids were also degraded with RNase and DNase. If LSPY01000004, LSPY01000006 were lysogenic or pseudolysogenic, their DNA should not have been found in the filtrate. Since all the colonies were processed separately, the presence of the same phage genomes in the filtrate from all the three termite colonies, suggests strongly that LSPY01000004 and LSPY01000006 enter an infective cycle. If ProJPt-1Bp1-like phages enter an infective cycle, studying their life cycle would aid in finding the mechanism by which intracellular phages penetrate two different types of cells, in this case the protozoa and its bacterial symbiont. It has been shown that *Wolbachia* phage WO can form virions and it has been suggested that phage WO can infect other *Wolbachia* cells from neighboring infected cells (Bordenstein et al., 2006; Kent and Bordenstein, 2010). The mechanism by which phage WO penetrates both the insect and bacterial cells remains elusive.

### LSPZ01000002 represents the genome of a phiKZ-like bacteriophage

The large contig LSPZ01000002 of 252,037 bp harboring a terminase gene was assembled from Colony 2. Further analysis of all the three termite colonies showed multiple contigs mapping against LSPZ01000002. Phylogenetic analysis of the terminase gene showed that LSPZ01000002 clustered with phiKZ-like bacteriophages (Figure 3). Of the predicted 238 genes, 71 genes had a match in the NCBI database with an e-value of 10^−5^ or less. Of those 71 genes, 35 genes matched to the phiKZ-like bacteriophage *Bacillus* phage AR9 (Lavysh et al., 2016). In all three termite colonies, *Bacillus* phage AR9-like genes were present in the highest abundance, suggesting that the bacteriophage, possibly along with its host has an important role in the termite gut. phiKZ-like bacteriophages encode two multi-subunit RNA polymerases (RNAPs); one of them is packed in the virion while the other is expressed in infected cells (Krylov et al., 2007; Ceyssens et al., 2014). These RNAPs are considered as the signature genes of phiKZ-like bacteriophages. LSPZ01000002 had six predicted genes encoding RNAP; three were predicted to encode the beta subunit, while three encoded the beta' subunit. Phylogenetic analysis of predicted virion (Figure S3) and non-virion RNAPs (data not shown) along with RNAPs from the other phiKZ-like phages produced a similar phylogenetic topology as described previously (Lavysh et al., 2016). LSPZ01000002 RNAPs were clustered with *Bacillus* phage AR9 and *Yersinia* phage phiR137.

LSPZ01000002 RNAPs were clustered with *Bacillus* phage AR9 and *Yersinia* phage phiR137. Comparative genomic analysis of LSPZ01000002, *Bacillus* phage AR9, and *Yersinia* phage phiR137 showed considerable similarities but no synteny in the genomes (Figure 4B). Lack of synteny in the genomes of closely-related phiKZ-like bacteriophages has been observed previously (Cornelissen et al., 2012; Jang et al., 2013). Most of the phiKZ-like bacteriophages are considered lytic in nature. However, some phages have been shown to be pseudolysogenic (Pletnev et al., 2010; Lavysh et al., 2016). Whether LSPZ01000002 is lytic or pseudolysogenic remains unknown. Unlike the genome of *Bacillus* phage AR9 which had multiple introns, RFAM BLAST did not predict any intron sequences in LSPZ01000002. One open reading frame (ORF) encoded a Group I intron-like endonuclease which showed similarities to many Group I introns from Firmicutes. One of the most important differences observed between Bacillus phage AR9 and LSPZ01000002 is the difference between ribonucleotide reductase (RNR) genes. Bacillus phage AR9 contains an operon of class I RNR genes (*nrdE and nrdF*). This class of RNR is dependent on oxygen and is found in organisms which can grow aerobically (Dwivedi et al., 2013). LSPZ01000002, on the other hand, contains an operon of class III RNR genes. This class is sensitive to oxygen and bacteriophages infecting strict anaerobes like *Clostridium* sp. harbor only this class of RNR genes (Dwivedi et al., 2013). The RNR genes found in LSPZ01000002 showed a high degree of similarity to *Treponema primitia* RNR genes, which is a strict anaerobic spirochete isolated from the gut of a damp wood termite *Zootermopsis angusticollis* (Graber et al., 2004). Many spirochetes have been previously reported from the gut of various termite species and some have been known to be ectosymbionts of the gut protozoa (Noda et al., 2003; Hongoh et al., 2007). These data suggest that LSPZ01000002 most likely infects a strict anaerobe from the termite gut, possibly a spirochete.

### LSPY01000009 and LSQA01000015 represent partial genomes of *lactococcus lactis* phage 1706-like phages

The three contigs LSPY01000009, LSPZ01000022, and LSQA01000015 were identified in termite Colony 1, 2, and 3 respectively. LSPY01000009 and LSPZ01000022 showed 99% similarity at genome level and hence were considered as genomes from the same phage. A moderate nucleotide level similarity was observed between LSPY01000009 and LSQA01000015 mostly at the ends of the two contigs (46% query coverage, 67% identity). All the three contigs showed a high degree of similarity to proteins from *Rhodococcus* phage ReqiPepy6 (Summer et al., 2011) and *Arthrobacter* phage Mudcat proteins. As observed in the *Rhodococcus* phage ReqiPepy6 and *Arthrobacter* phage Mudcat, LSQA01000015 lacked reverse transcriptase in the genome. However, LSPY01000009 and LSPZ01000022 harbored a reverse transcriptase enzyme belonging to Group II introns. Another important difference observed between the three termite gut contigs, *Rhodococcus* phage ReqiPepy6 and *Arthrobacter* phage Mudcat, is the presence of anaerobic ribonucleotide reductase gene (*nrdD*). The *nrdD* gene was present in all the three termite gut contigs but not in ReqiPepy6 and Mudcat. The *nrdD* gene has been previously observed in the genomes of bacteriophages infecting anaerobes (Dwivedi et al., 2013). No other class of RNR gene was observed in any of the contigs. Interestingly, RNR genes in LSPY01000009 and LSQA01000015 showed very little similarity to each other at amino acid level. Comparative genomic analysis showed segments of synteny in structural, DNA metabolism-related genes and segments of variable small hypothetical proteins (Figure S4). It has been shown that *Rhodococcus* phage ReqiPepy6 along with other closely related phages from *Lactococcus lactis* phage 1706-like phages have segments of genome expansion (Summer et al., 2011). In that case, closely related bacteriophages have segments of conserved genes but differ from each other in genome segments where multiple small hypothetical proteins are observed (Lavigne et al., 2009). Another characteristic of *L. lactis* phage 1706-like phages is the enrichment of membrane related proteins (14–23%) (Garneau et al., 2008; Summer et al., 2011).

In all the three contigs, between 20 and 23% of the predicted proteins were found to contain at least one transmembrane domain. Based on this analysis, LSPY01000009, LSPZ01000022, and LSQA01000015 represent partial genomes of *L. lactis* phage 1706-like phages which most likely infects a Firmicutes bacterium.

### LSPZ01000027 represents a full genome of a temperate phage

LSPZ01000027 was identified as a circular contig in termite Colony 2. Other multiple contigs could be mapped against LSPZ01000027 from all the three termite colonies. LSPZ01000027 genome showed high level of synteny to structural genes of *Clostridium* phage phiCDHM1 (Hargreaves et al., 2014) and *Clostridium* phage phiMMP01 (Boudry et al., 2015) (Figure S4). PhiCDHM1 genome was found to harbor a cassette of bacterial quorum sensing genes (Hargreaves et al., 2014), but no such cassette was identified in LSPZ01000027. PhiCDHM1 is considered a member of phiCD119-like bacteriophages with the presence of a signature DNA replication cassette. In LSPZ01000027 this signature DNA replication cassette was not observed. Also the G+C content of LSPZ01000027 was much higher (42%) than that of phiCDHM1 and phiMMP01the (G+C content 14–29%). Similar to the genome of phiCDHM1 and phiMMP01, the genome of LSPZ01000027 has many proteins annotated as putative anti-repressor proteins. The true identity of these anti-repressor proteins remains unknown at this moment. LSPZ01000027 also had a Group II intron encoded reverse transcriptase. The presence of an integrase and multiple anti-repressor-like proteins indicate that LSPZ01000027 is most likely a temperate bacteriophage.

### LSQA01000020 represents the genome of a lytic siphovirus

LSQA01000020 was identified as a linear contig in termite Colony 3 and multiple contigs from Colony 2 could be mapped against LSQA01000020. The first and the last gene of the linear contig encoded the same partial gene, indicating an almost complete circular bacteriophage genome. Phylogenetic analysis of the terminase gene from LSQA01000020 placed it in the Bacteroidetes cluster in a sister clade with *Flavobacter* bacteriophages. Comparative genomic analysis of the LSQA01000020 genome shows a high level of similarity to the genomes of two bacteriophages, P2559S and P2559Y, both of which infect the Bacteroidetes species *Croceibacter atlanticus* (Kang et al., 2012, 2016). Even though both P2559S and P2559Y are lytic Siphoviruses infecting the same species they show similarity only in the structural module of the genome (Kang et al., 2016). LSQA01000020 showed similarity in the structural module to both the phages and similarity to some extent in the replication module to P2559Y (Figure S4). LSQA01000020 contained thymidylate synthase and asparagine synthase genes, which were absent in P2559S and P2559Y. Based on the similarity to P2559S and P2559Y and phylogenetic placement of the terminase gene, LSQA01000020 most likely infects a Bacteroidetes species.

Contigs similar to the first bacteriophage (CVT22) isolated from the termite gut were also observed in Colony 3 suggesting some association with the termite gut (Tikhe et al., 2015). It has been suggested that CVT22 may represent a founding member of a new cluster of lytic bacteriophages (Casjens and Grose, 2016) and the termite gut might represent a niche of diversity of CVT22-like bacteriophages.

Many contigs observed in our virome, harbored signature phage genes. Large contigs of 200,000 bp or more were observed in all the three termite colonies. However, due to the presence of a large proportion of previously unknown genes, the origin of many contigs remains unclear. As more phage genomes will be sequenced to populate reference databases we believe that many more phage genomes will be uncovered from the termite gut.

Large viruses like the Chlorella virus infecting the algal symbionts of the protozoa have been previously identified and sequenced (Yamada et al., 2006). In our study, we found ORFs similar to large eukaryotic dsDNA viruses. However, on further analysis we could not confidently assign the origin of these genes as viral. Further research is required to study the presence of large eukaryotic ds-DNA viruses from the termite gut.

### Termite gut microviruses represent a putative new sub-family

As compared to double-stranded DNA viruses, single-stranded DNA (ssDNA) viruses were present in a lower abundance (1–10%). Colony 1 had the most diverse community of ssDNA viruses, with genes from 38 different types of ssDNA viruses followed by Colony 2 (26 types) and Colony 3 (15 types). Most of the dominant ssDNA viruses were conserved in all the three colonies along with some inter-colonial differences (Figure S5).

**Figure 5:**
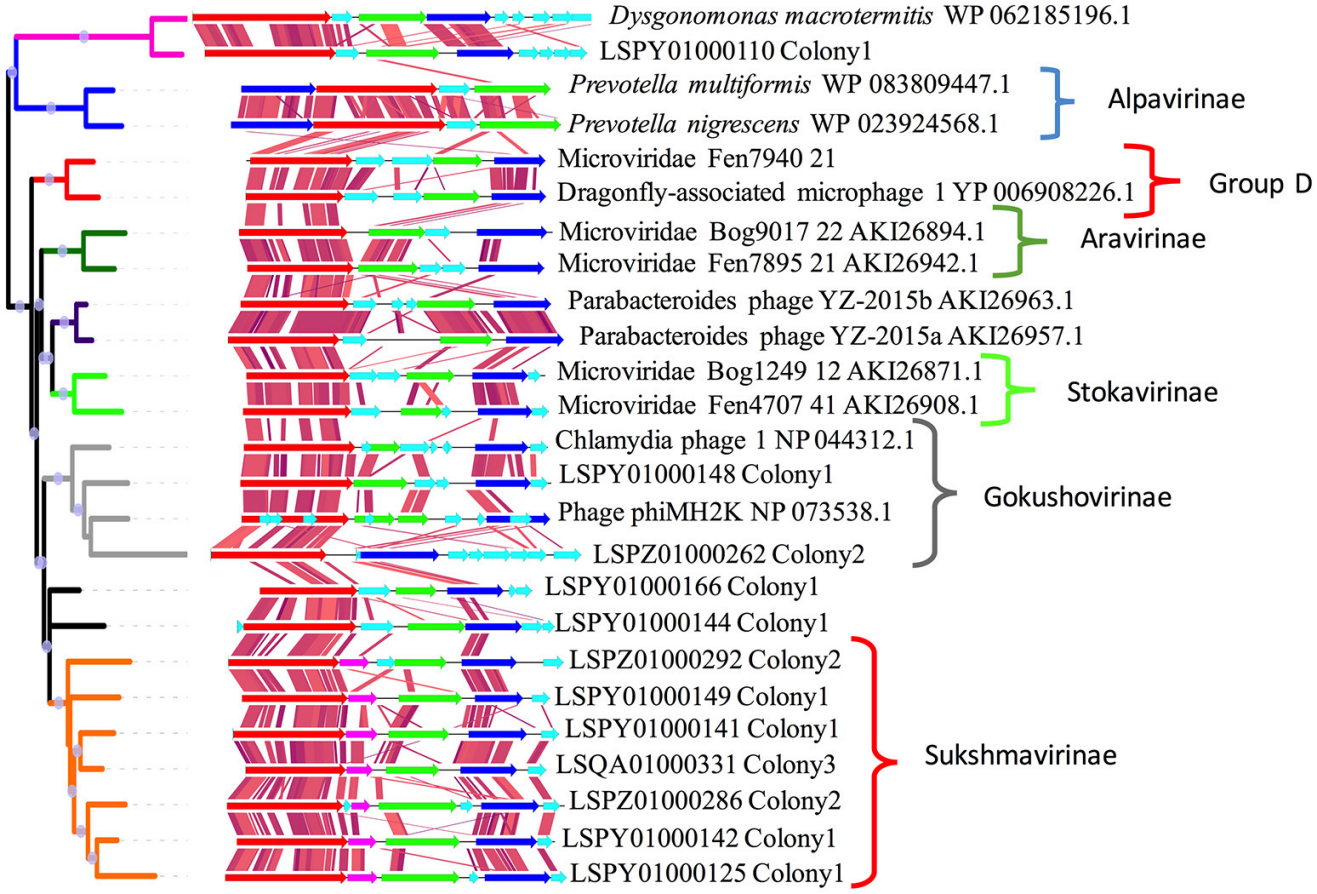
A maximum likelihood phylogenetic tree of Microviridae VP1 from the guts of workers from three termite colonies along with the comparative genomic analysis of closely related Microviruses. Nodes with a bootstrap score of more than 70% are indicated by gray circles. For this figure, only VP1 genes from putative full Microviridae genomes were used. VP1 is colored red, VP2: green, VP4: blue. An extra ORF found after the VP1gene of Sukshmaviriane is colored pink.

Phylogenetic analysis of VP1 major capsid gene indicated a diverse population of Microviridae in the termite gut. We were able to construct 12 novel complete genomes of Microviridae from all three termite colonies. Phylogenetically, VP1 from LSPY01000110 was placed in a cluster with *Dysgonomonas macrotermitis*, a bacterium of the phylum Bacteroidetes, which was previously isolated from the gut of a higher termite (*Macrotermes barneyi*) (Yang et al., 2014). Bacteria of this genus are also known to be a part of the gut community of the FST (Husseneder et al., 2009, 2010). LSPY01000110 showed synteny to a contig from the genome of a *D. macrotermitis* (Figure 5). It has been previously reported that Microviridae bacteriophage can undergo a temperate life cycle in Bacteroidetes (Krupovic and Forterre, 2011). The *D. macrotermitis* prophage-like sequence and LSPY01000110 showed the same gene order (VP1-ORF2-VP2-VP4) followed by five ORFs encoding hypothetical proteins in *D. macrotermitis* and four ORFs in case of LSPY01000110. The hypothetical proteins showed no similarity to each other. Although LSPY01000110 and *D. macrotermitis* prophage were clustered with the Alpavirinae subfamily, comparative genomics showed very little similarity between the two clusters.

Phylogenetic analysis of the VP1 sequences of LSPZ01000262 and LSPY01000148 placed them in the Gokushovirinae subfamily (Figure 5). The genome arrangements of LSPZ01000262 and LSPY01000148 were different from previously described Gokushovirinae sequences (Quaiser et al., 2015). No clear distinguishable VP5 sequences were observed in LSPZ01000262 and LSPY01000148.

VP1 from LSPY01000144 and LSPY01000166 could not be confidently placed in any cluster. VP1 from seven contigs formed a completely separate cluster from all of the other Microviridae. The genome arrangement also showed a conserved order (VP1-ORF1-VP2-VP4-ORF2). In this cluster, the ORF present after theVP1 gene encoded for a hypothetical protein. This protein did not match any known protein in the NCBI database but showed a high similarity to proteins in the cluster. The protein encoded by the ORF after VP4 from some contigs from the cluster showed similarity to Gokushovirinae VP5. Based on the genome arrangement and the VP1 phylogeny, we propose a new subfamily Sukshmavirinae (Sukshma is the Sanskrit word for “small”) for the sequences observed in the termite gut virome.

### A diverse community of circoviruses is present in the termite gut

Circoviruses are small ssDNA viruses known to infect a number of higher eukaryotes (Todd et al., 2001). In recent years Circovirus-like genomes have been identified to be associated with a variety of animals including many insects (Rosario et al., 2011; Garigliany et al., 2015). The exact role of Circoviruses associated with various animals is currently not understood completely.

**Figure 6:**
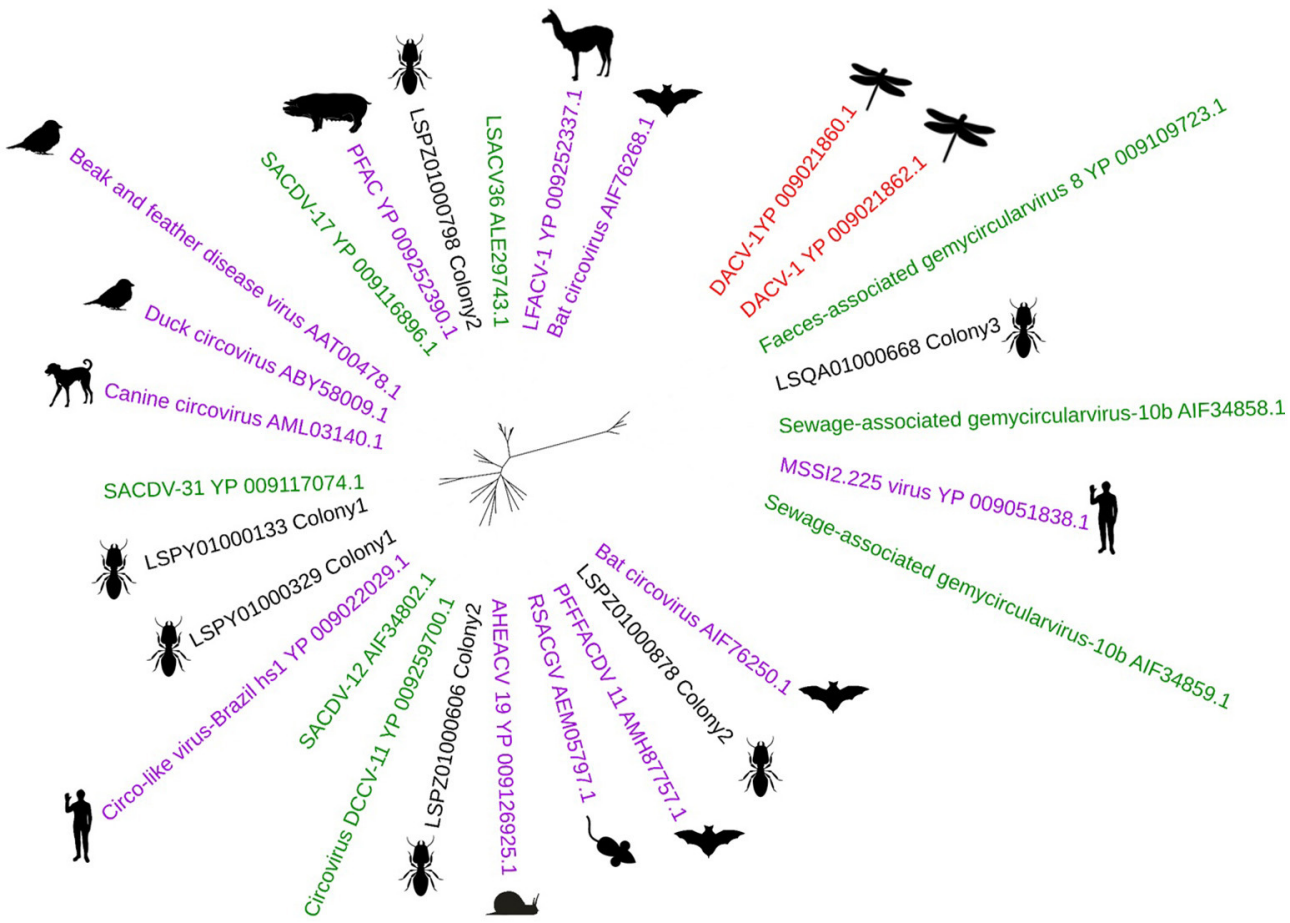
Unrooted maximum likelihood phylogenetic tree of Circoviridae replication initiation protein. The hosts of the Circoviruses are displayed in a picture next to the sequence. Environmental Circoviruses are shown in green. Insect related Circoviruses are shown in red.

From all three termite colonies, 10 novel Circoviridae-like genomes were assembled. The genome size ranged between 1,388 and 5,851 bp. All the genomes encoded the Circoviridae Rep protein which is considered as the signature gene of the family. Phylogenetic analysis of the Rep gene showed two distinct groups, one belonging to the Cycloviruses and the other to the Circoviruses (Figure 6). There was no correlation between the host of these viruses and the phylogenetic placement of the Rep proteins. Termite gut Rep proteins were distributed all over the phylogenetic tree with only one sequence clustered in the Cyclovirus group.

So far, there have been very limited data on viruses capable of infecting termites (Al Fazairy and Hassan, 1988). It has been suggested that a virus infecting termites would be an ideal candidate for biological control (Chouvenc et al., 2011). The origin of the Circoviruses found in the termite gut remains unclear. Whether these viruses are environmental and were ingested by the termites or they actually infect the termites is an area of future research.

### Functional annotation shows a high degree of conservation in the termite gut virome

MG-RAST functional analysis assigned functions to 14.5% of the predicted proteins from Colony 1, 27.62% from Colony 2 and 33.17% from Colony 3. These results show that the termite virome is largely comprised of proteins whose function still remains unknown. Functional annotation of the assigned genes indicated that phage, prophages, transposable elements, and plasmids were present in the highest abundance in all the three termite colonies (Figure S6).

The presence of conserved sequences was expected because most of the bacteriophages carry out the same core basic functions such as replication, lysis, packaging, and host integration during their life cycle. To study the conservation of functional genes in the gut virome, we analyzed the orthologous gene clusters present in the termite colonies. Orthologous genes may represent a difference in the sequence but the function is generally conserved. The orthovenn analysis of the gut virome from all the three termite colonies showed that all the 53,000 protein sequences formed a total of 9,625 clusters. Of these clusters, 8,317 orthologous clusters contained genes from at least two of the three termite colonies. A total of 3,823 clusters comprised of 12,295 protein sequences were shared by all three colonies. The data suggest that the FST gut virome we sequenced, has a core set of functional genes that is conserved between all the three termite colonies.

### Termites possibly harbor a core virome comprised of bacteriophages infecting protozoal endosymbiotic bacteria

The FST is native to China and was introduced into the USA (Hawaii) over a century ago (Husseneder et al., 2012). The first recorded report of FSTs on the US mainland is from 1957 (Chambers et al., 1988). A previous study showed that the bacterial composition of the FST gut between the native (China) and the introduced (New Orleans, USA) populations did not change significantly (Husseneder et al., 2010). The obligatory symbionts of the gut protozoa form the core of the conserved bacterial community of the FST gut (Noda et al., 2005). These symbionts are found exclusively in the termite gut. In this study we reported two bacteriophages LSPY01000004 and LSPY01000006, infecting the obligatory endosymbiotic bacteria. The similarity between LSPY01000004, LSPY01000006, and phage ProJPt-1Bp1 is remarkable despite the fact that they were sequenced independently at different time points in geographically well-separated areas (USA and Japan) and from two different species of termites. It has been speculated that phage related genes might be ubiquitous in the termite gut (Tadmor et al., 2011). In that study, closely related bacteriophage genes were found in different species of termites collected almost 20 years apart in two different geographic locations (Tadmor et al., 2011). Interestingly, we have also found LSPY01000004 genes in other FST gut samples collected from additional colonies in New Orleans at different time points (unpublished data).

A core virome has been previously observed in human saliva and lower respiratory tract (Willner et al., 2009; Pride et al., 2012). In a study of human virome, bacteriophage crAssphage related sequences were found globally distributed in many human fecal metagenome samples (Dutilh et al., 2014). Based on the taxonomic and functional overlap of phages among the three different termite colonies evidenced by shared phages and gene functions, we hypothesize that termites also harbor a highly conserved core virome. We are aware that the main limitation of this study is a small sample size from the same geographic area. It cannot be ruled out that similar environmental conditions may be responsible for observed similarities in the virome of the three termite colonies. It should be noted that at this point the “termite core virome” is a hypothesis and including more termite samples from various geographical areas will help to corroborate or refute our hypothesis.

Phylogenetic analysis of the terminase and integrase genes indicated that termite gut viruses show a high degree of similarity to prophage genes from diverse bacteria. Termites are highly dependent on their gut bacteria to complement their own metabolism, and changes in the bacterial population have been shown to negatively affect the termite host (Rosengaus et al., 2011; Brune, 2014). The impact of bacteriophage pressure on the bacteria is not only known to alter species composition but also their metabolic processes (Middelboe et al., 2009). It would be essential for the termite and the gut bacteria to maintain a functionally conserved set of biochemical pathways despite the presence of bacteriophage pressure. Hence, it would be advantageous to the termite host and the symbiotic gut community upon which it relies, if the bacteriophage adopts a temperate life style rather than lytic one. In turn, the phage replicates with a thriving bacterial host population and is transferred throughout the termite colony via social interactions. Manipulating the bacterial host abundance and studying the bacteriophage dynamics would further help us understand the lytic-lysogenic life style changes in the termite gut.

Previous studies have shown that temperate bacteriophages can protect the bacterial host from other bacteriophages by superinfection immunity (Bondy-Denomy and Davidson, 2014). Termites are soil dwelling and the gut bacteria must be encountering a number of environmental bacteriophages. It would be interesting to study whether termite gut bacteriophages prevent the gut bacteria from environmental bacteriophages via superinfection immunity. In the future, we intend to develop termites as a model system to study the complicated quadripartite relationship between bacteria, bacteriophages, gut protozoa, and the termites themselves.

## Author contributions

CT designed and conducted the experiments and wrote the manuscript, CH designed the experiments, supervised the project, and edited the manuscript.

### Conflict of interest statement

The authors declare that the research was conducted in the absence of any commercial or financial relationships that could be construed as a potential conflict of interest.

## Supplementary material

Supplementary Table 1 Table describing the characteristics of predicted tailed bacteriophage genomes. *LSPY01000009, LSPZ01000022 and LSQA01000015 represent similar bacteriophages. Only data for LSPY01000009 is shown here.

Figure S1 A maximum likelihood phylogenetic tree of large terminase subunit of type terminase_3 **(A)**, terminase_GPA **(B)**, terminase_1 **(C)**. The nodes with a bootstrap value of 70% or more are indicated by a circular symbol. Sequences from the termite gut are colored purple. Bacteriophages: red. Firmicutes: orange. Spirochetes: dark blue. Gammaproteobacteria: bright green. Bacteroidetes: dark green. Alphaproteobacteria: gray. Others: black.

Figure S2 A maximum likelihood phylogenetic tree of phage integrase genes. The nodes with a bootstrap value of 70% or more are indicated by a circular symbol. Sequences from the termite gut are colored purple Firmicutes: orange. Actinobacteria: red. Spirochetes: dark blue. Gammaproteobacteria: bright green. Bacteroidetes: dark green. Planctomycetes: sky blue. Others: black.

Figure S3 Unrooted maximum likelihood phylogenetic tree of RNAP beta subunit of virion and non-virion subunits from phiKZ-like bacteriophages. RNAPs from LSPZ01000002 are shown in purple. RNAP from *Thermus thermophilus* is used as an outgroup.

Figure S4 **(A)** Comparative genomic analysis of LSPY01000009 and LSQA01000015 with *Arthrobacter* phage Mudcat and *Rhodococcus* phage ReqiPepy6. **(B)** Comparative genomic analysis of LSPZ01000027 with *Clostridium* phage cdhm1 and *Clostridium* phage phimmp01. **(C)** Comparative genomic analysis of LSQA01000020 with *Croceibacter* phage P2559S and *Croceibacter* phage P2559Y. All the genomes have been rearranged so that the start codon of the large terminase subunit is the first base in the sequence. The figures were generated using Easyfig software with tblastx. The structural genes are indicated in red color, DNA metabolism related genes in blue, cell lysis in green, virulence associated protein in pink, yellow anti-repressor, reverse transcriptase in purple. All the other genes are indicated in sky blue color.

Figure S5 Taxonomic distribution (%) of the predominant ssDNA bacteriophage genes (normalized) from the guts of the termites from three different colonies. The data were generated using Metavir-2 server by comparing the predicted proteins to NCBI virus protein database. Top BLAST hits with an e-value of 10^−5^ or less were used.

Figure S6 Normalized percent distribution of the functional categories of predominant genes from the guts of the termites from three different colonies. The data were generated using MG-RAST server by comparing the predicted proteins to MD5nr database.
