# Supplementary material for: Metavirome Sequencing of the Termite Gut Reveals the Presence of an Unexplored Bacteriophage Community
Source: Front Microbiol. 2018 Jan 4;8:2548. doi: 10.3389/fmicb.2017.02548 (PMC5759034; doi:10.3389/fmicb.2017.02548)
Supplement: Supplementary Table 1 — Table describing the characteristics of predicted tailed bacteriophage genomes. *LSPY01000009, LSPZ01000022 and LSQA01000015 represent similar bacteriophages. Only data for LSPY01000009 is shown here. [file Table1.pdf]

Supplementary table 1

Contig	Size	Coverage (X)	No. of genes	Type	Match to viral database (e-value 10^{-5} or less) No. of genes	Match to PfamA database (e-value 10^{-5} or less) No. of genes	Match to POG13 database (e-value 10^{-5} or less) No. of genes	Virfam family assignment
LSPY01000009*	49,532	16.61	59	Linear	34 (57.62 %)	14 (23.72%)	24 (40.67 %)	Siphoviridae
LSPZ01000027	43,096	21.66	59	Circular	41(69.49 %)	29 (17.11%)	35 (59.32 %)	Myoviridae
LSPZ01000002	2,52,037	35.68	230	Linear	40 (17.39 %)	26 (11.30%)	43 (18.69 %)	Myoviridae
LSQA01000020	44,285	12.99	51	Linear	44 (86.27 %)	22 (43.13%)	19 (37.25 %)	Siphoviridae
LSPY01000004	1,00,499	445.19	67	Circular	21 (31.34%)	5 (7.46 %)	4 (5.97 %)	No
LSPY01000006	98,046	63.81	65	Circular	25 (38.46%)	6.9 (23 %)	4 (6.15 %)	No

Supplementary table 1: Table describing the characteristics of predicted tailed bacteriophage genomes. * LSPY01000009, LSPZ01000022 and LSQA01000015 represent similar bacteriophages. Only data for LSPY01000009 is shown here
