# Supplementary figures and images for: Metavirome Sequencing of the Termite Gut Reveals the Presence of an Unexplored Bacteriophage Community
Source: Front Microbiol. 2018 Jan 4;8:2548. doi: 10.3389/fmicb.2017.02548 (PMC5759034; doi:10.3389/fmicb.2017.02548)

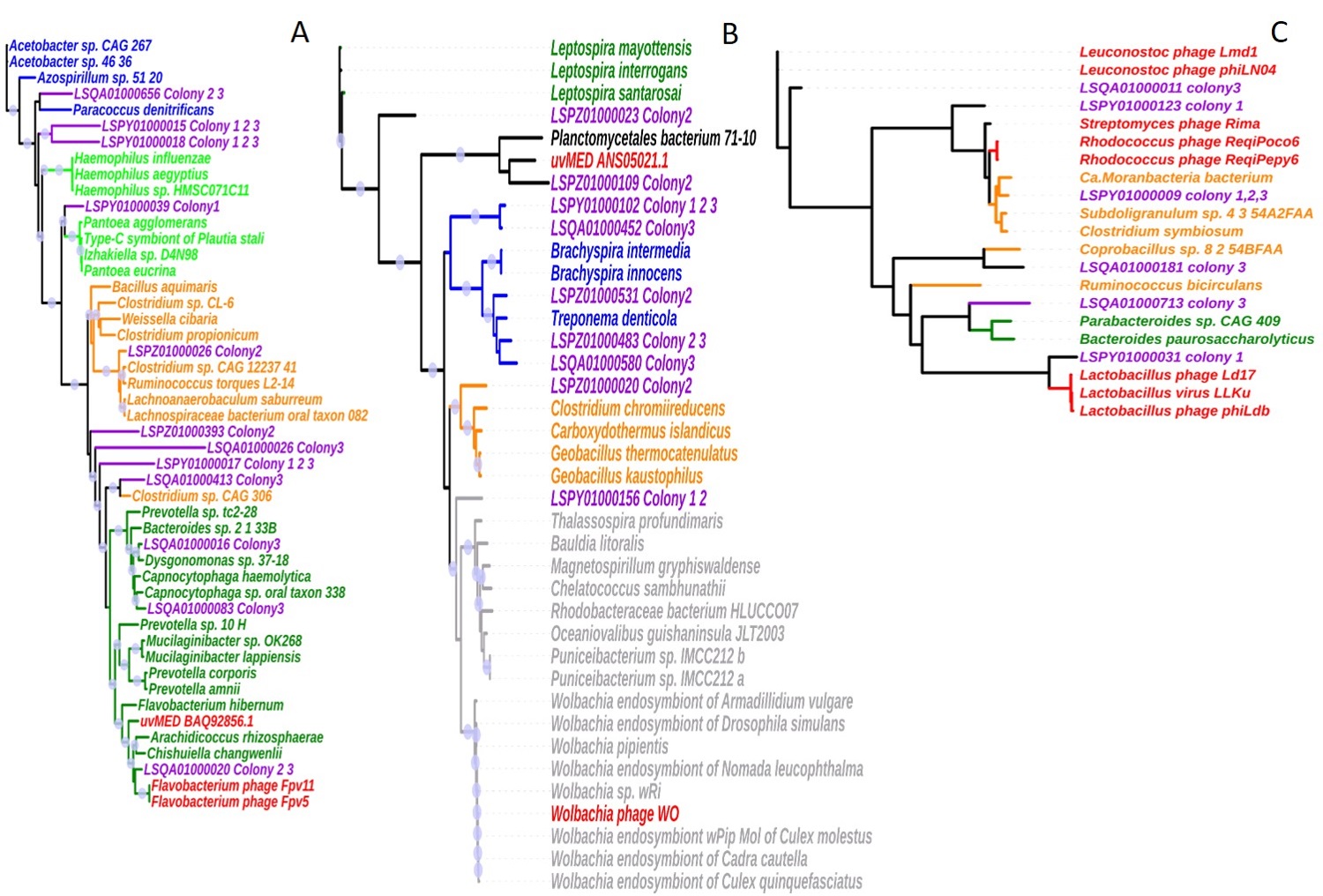

Supplement: Figure S1 — A maximum likelihood phylogenetic tree of large terminase subunit of type terminase_3 (A), terminase_GPA (B), terminase_1 (C). The nodes with a bootstrap value of 70% or more are indicated by a circular symbol. Sequences from the termite gut are colored purple. Bacteriophages: red. Firmicutes: orange. Spirochetes: dark blue. Gammaproteobacteria: bright green. Bacteroidetes: dark green. Alphaproteobacteria: gray. Others: black. [file Image1.JPEG]

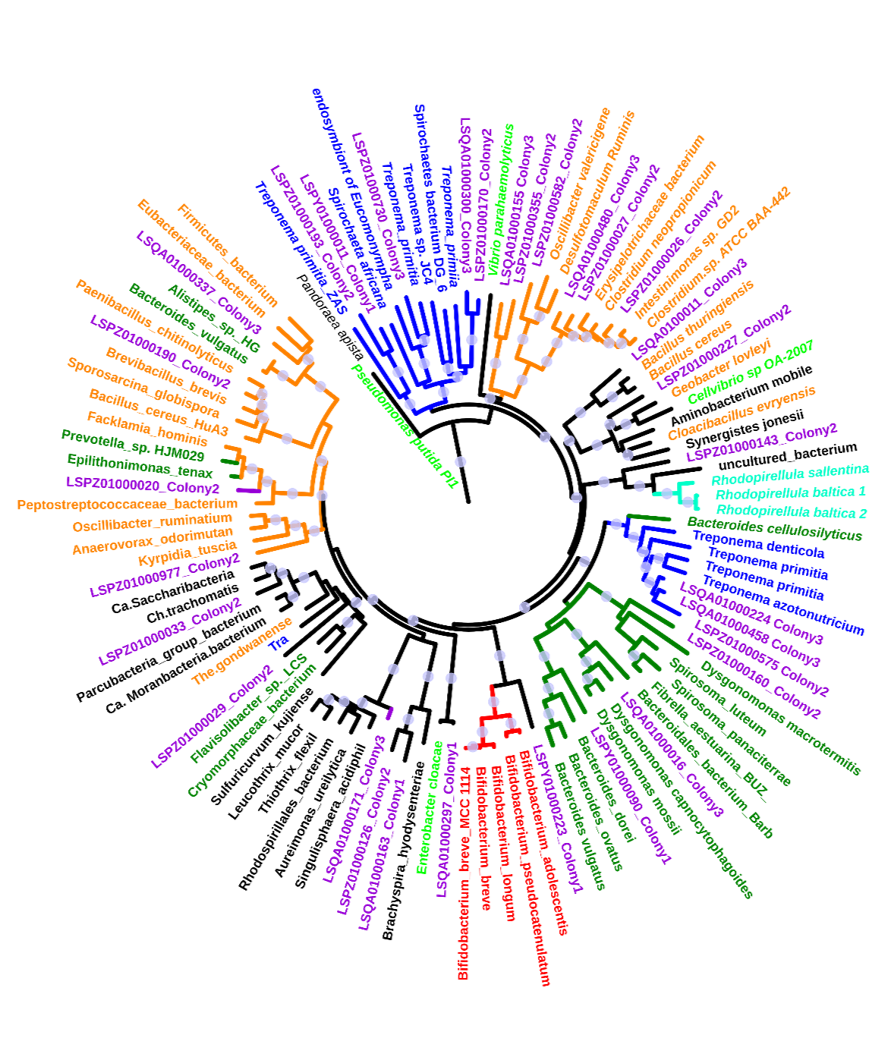

Supplement: Figure S2 — A maximum likelihood phylogenetic tree of phage integrase genes. The nodes with a bootstrap value of 70% or more are indicated by a circular symbol. Sequences from the termite gut are colored purple Firmicutes: orange. Actinobacteria: red. Spirochetes: dark blue. Gammaproteobacteria: bright green. Bacteroidetes: dark green. Planctomycetes: sky blue. Others: black. [file Image2.TIF]

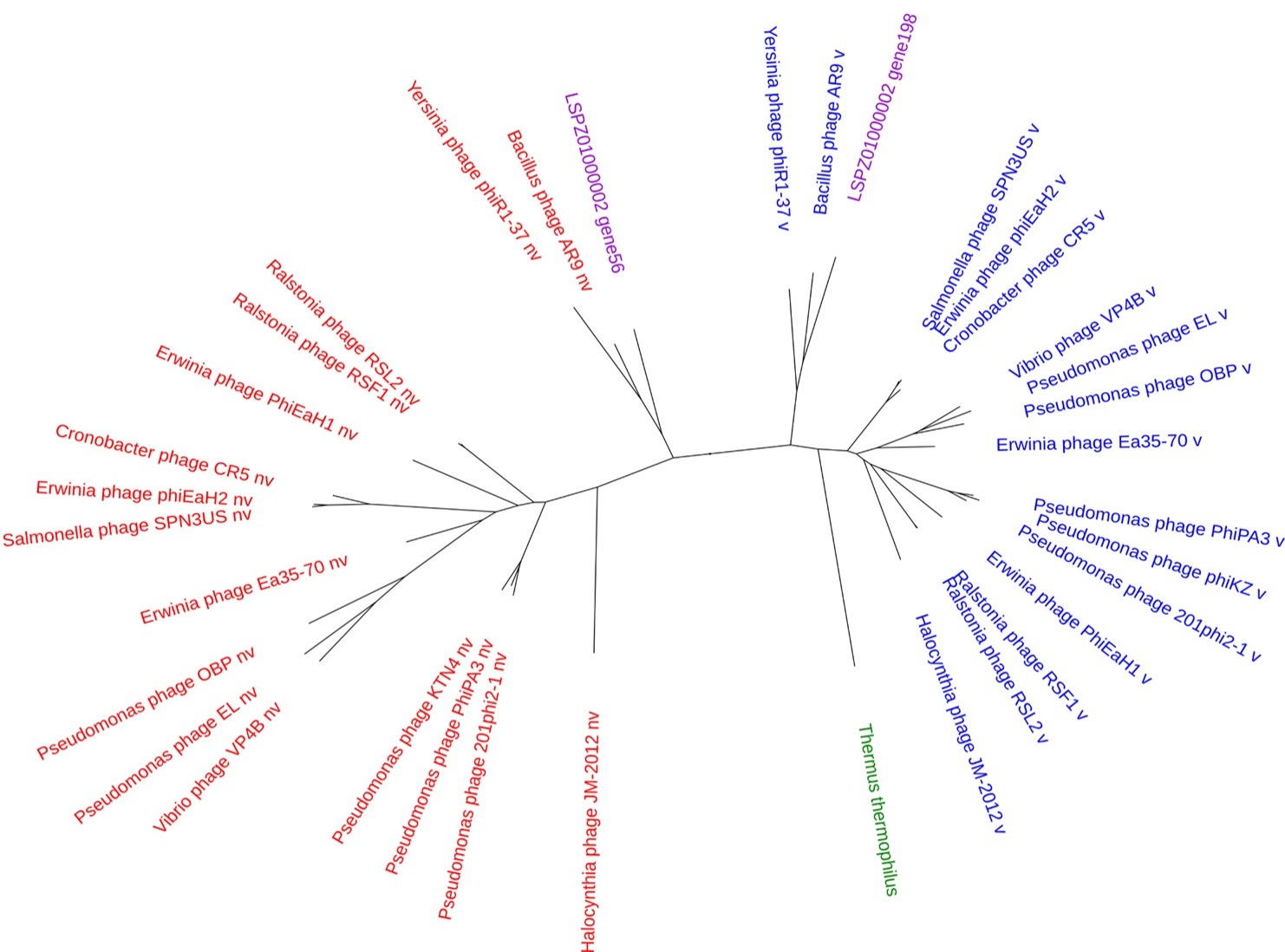

Supplement: Figure S3 — Unrooted maximum likelihood phylogenetic tree of RNAP beta subunit of virion and non-virion subunits from phiKZ-like bacteriophages. RNAPs from LSPZ01000002 are shown in purple. RNAP from Thermus thermophilus is used as an outgroup. [file Image3.JPEG]

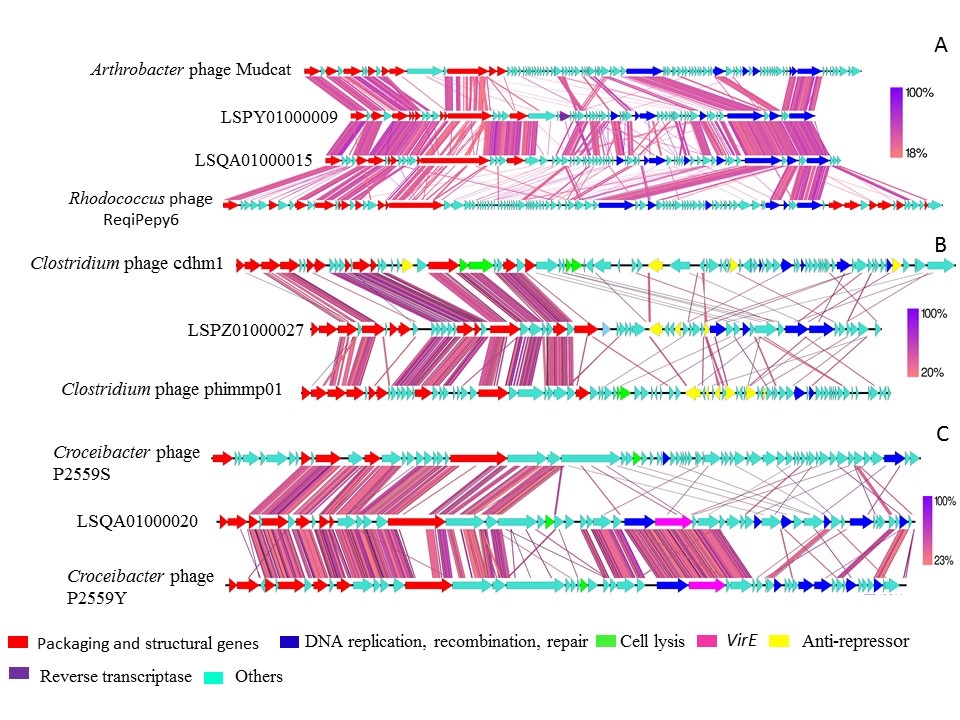

Supplement: Figure S4 — (A) Comparative genomic analysis of LSPY01000009 and LSQA01000015 with Arthrobacter phage Mudcat and Rhodococcus phage ReqiPepy6. (B) Comparative genomic analysis of LSPZ01000027 with Clostridium phage cdhm1 and Clostridium phage phimmp01. (C) Comparative genomic analysis of LSQA01000020 with Croceibacter phage P2559S and Croceibacter phage P2559Y. All the genomes have been rearranged so that the start codon of the large terminase subunit is the first base in the sequence. The figures were generated using Easyfig software with tblastx. The structural genes are indicated in red color, DNA metabolism related genes in blue, cell lysis in green, virulence associated protein in pink, yellow anti-repressor, reverse transcriptase in purple. All the other genes are indicated in sky blue color. [file Image4.JPEG]

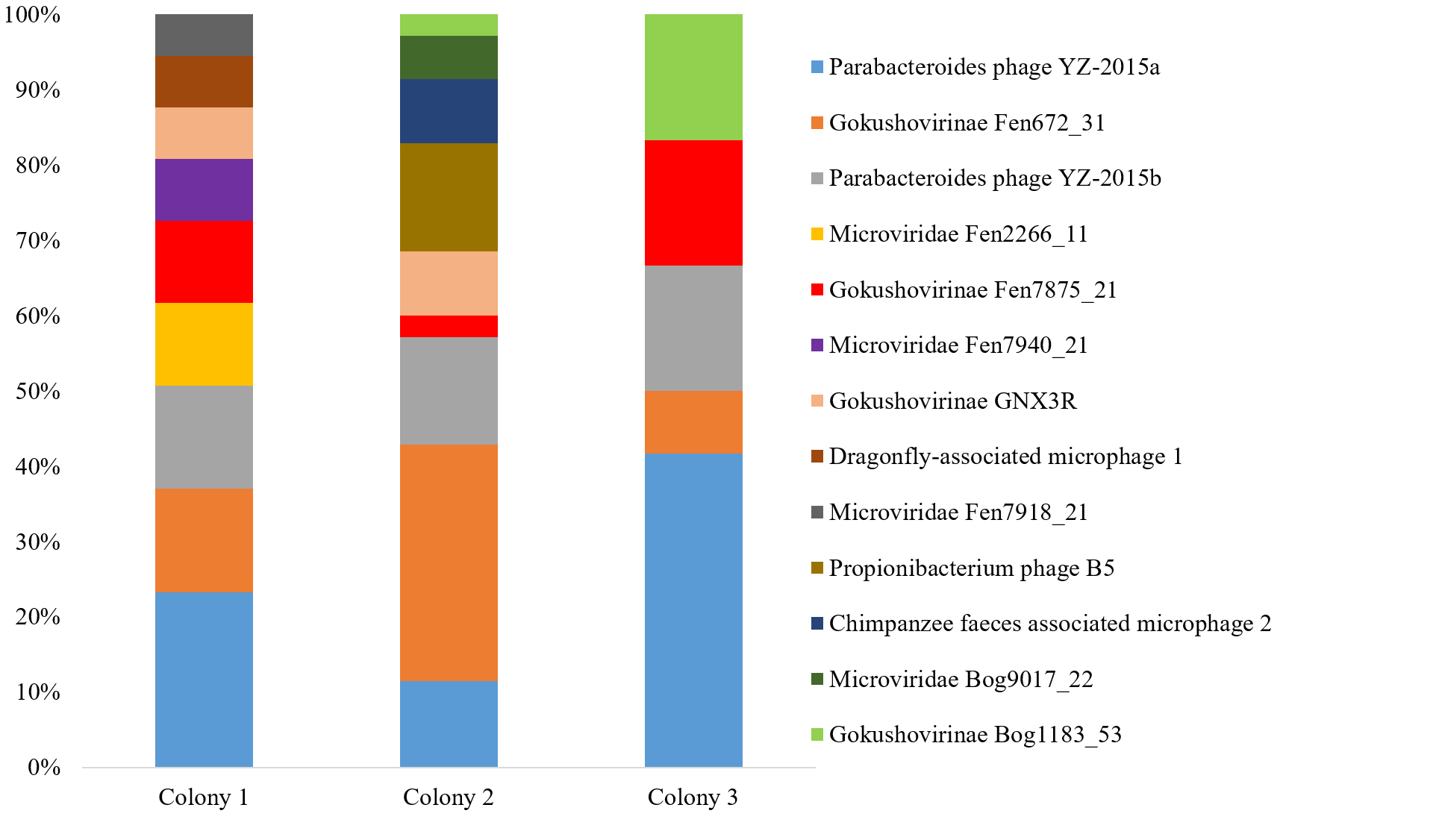

Supplement: Figure S5 — Taxonomic distribution (%) of the predominant ssDNA bacteriophage genes (normalized) from the guts of the termites from three different colonies. The data were generated using Metavir-2 server by comparing the predicted proteins to NCBI virus protein database. Top BLAST hits with an e-value of 10−5 or less were used. [file Image5.TIF]

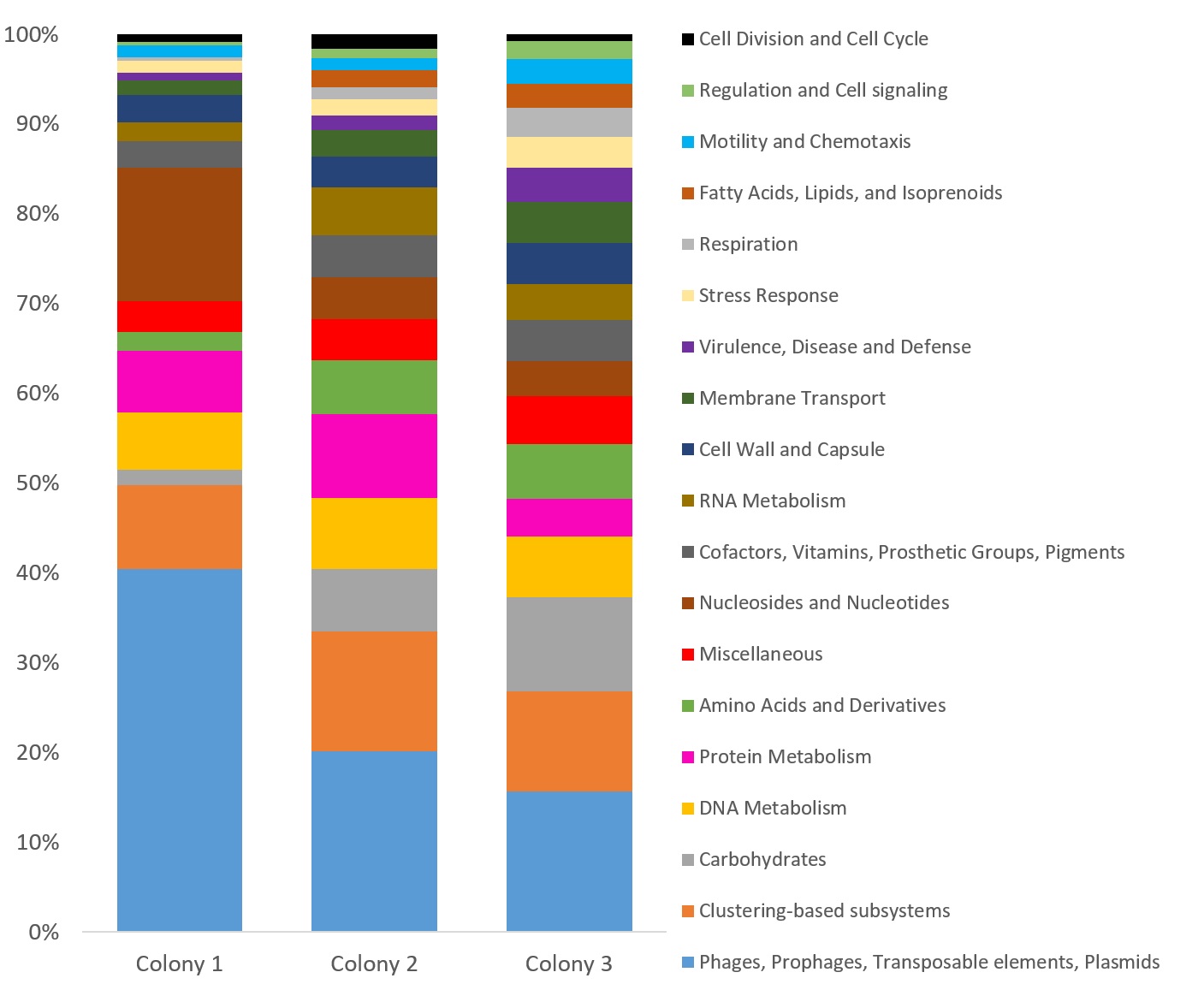

Supplement: Figure S6 — Normalized percent distribution of the functional categories of predominant genes from the guts of the termites from three different colonies. The data were generated using MG-RAST server by comparing the predicted proteins to MD5nr database. [file Image6.JPEG]
